# Injection fears and COVID-19 vaccine hesitancy

**DOI:** 10.1017/S0033291721002609

**Published:** 2021-06-11

**Authors:** Daniel Freeman, Sinéad Lambe, Ly-Mee Yu, Jason Freeman, Andrew Chadwick, Cristian Vaccari, Felicity Waite, Laina Rosebrock, Ariane Petit, Samantha Vanderslott, Stephan Lewandowsky, Michael Larkin, Stefania Innocenti, Helen McShane, Andrew J. Pollard, Bao Sheng Loe

**Affiliations:** 1 Department of Psychiatry, University of Oxford, Oxford, UK; 2 Oxford Health NHS Foundation Trust, Oxford, UK; 3 NIHR Oxford Health Biomedical Research Centre (BRC), Oxford, UK; 4 Nuffield Department of Primary Care, University of Oxford, Oxford, UK; 5 Department of Communication and Media, Online Civic Culture Centre, Loughborough University, Loughborough, UK; 6 Oxford Vaccine Group, Department of Paediatrics, University of Oxford, Oxford, UK; 7 School of Psychological Science, University of Bristol, Bristol, UK; 8 Department of Psychology, Life and Health Sciences, Aston University, Birmingham, UK; 9 Smith School of Enterprise and the Environment, University of Oxford, Oxford, UK; 10 The Jenner Institute, Nuffield Department of Medicine, University of Oxford, Oxford, UK; 11 NIHR Oxford Biomedical Research Centre (BRC), Oxford, UK; 12 The Psychometrics Centre, University of Cambridge, Cambridge, UK

**Keywords:** COVID-19 vaccine hesitancy, needle fears, blood-injection-injury phobia, UK adults

## Abstract

**Background:**

When vaccination depends on injection, it is plausible that the blood-injection-injury cluster of fears may contribute to hesitancy. Our primary aim was to estimate in the UK adult population the proportion of COVID-19 vaccine hesitancy explained by blood-injection-injury fears.

**Methods:**

In total, 15 014 UK adults, quota sampled to match the population for age, gender, ethnicity, income and region, took part (19 January–5 February 2021) in a non-probability online survey. The Oxford COVID-19 Vaccine Hesitancy Scale assessed intent to be vaccinated. Two scales (Specific Phobia Scale-blood-injection-injury phobia and Medical Fear Survey–injections and blood subscale) assessed blood-injection-injury fears. Four items from these scales were used to create a factor score specifically for injection fears.

**Results:**

In total, 3927 (26.2%) screened positive for blood-injection-injury phobia. Individuals screening positive (22.0%) were more likely to report COVID-19 vaccine hesitancy compared to individuals screening negative (11.5%), odds ratio = 2.18, 95% confidence interval (CI) 1.97–2.40, *p* < 0.001. The population attributable fraction (PAF) indicated that if blood-injection-injury phobia were absent then this may prevent 11.5% of all instances of vaccine hesitancy, AF = 0.11; 95% CI 0.09–0.14, *p* < 0.001. COVID-19 vaccine hesitancy was associated with higher scores on the Specific Phobia Scale, *r* = 0.22, *p* < 0.001, Medical Fear Survey, *r* = 0.23, *p* = <0.001 and injection fears, *r* = 0.25, *p* < 0.001. Injection fears were higher in youth and in Black and Asian ethnic groups, and explained a small degree of why vaccine hesitancy is higher in these groups.

**Conclusions:**

Across the adult population, blood-injection-injury fears may explain approximately 10% of cases of COVID-19 vaccine hesitancy. Addressing such fears will likely improve the effectiveness of vaccination programmes.

## Introduction

Vaccination is now a key global public health intervention to combat the severe acute respiratory syndrome coronavirus 2 (SARS-CoV-2) pandemic. A variable proportion of the population in each country will seek to delay or avoid being vaccinated, thereby limiting the success of vaccination programmes. In the Oxford Coronavirus Explanations, Attitudes, and Narratives Survey II (OCEANS-II), key factors associated with vaccine hesitancy in the UK population were lower perception of risk from the virus, less awareness of the collective benefits of vaccination, doubts about the efficacy of vaccination and worry about potential side effects, particularly in the context of the rapid development and testing of the vaccines (Freeman et al., 2020). In subsequent qualitative interviews, participants raised an issue that had not been assessed in the survey: anxiety about injections. Fears about injection in the short term might prevent the long-term benefits of vaccination. In children, adolescents and adults, fear of injection falls within the unitary specific phobia subtype of blood-injection-injury fears (Kendler, Aggen, Werner, & Fried, 2020; Loken, Hettema, Aggen, & Kendler, 2014; Muris, Schmidt, & Merckelbach, 1999; Wenzel & Holt, 2003). This is persistent excessive fear of blood, needles or invasive medical procedures, leading to avoidance or endurance with intense anxiety. Uniquely among anxiety disorders, part of the typical response pattern for this phobia is a drop in heart rate and blood pressure leading to fainting. A fear of fainting may cause great reluctance to join a line of people waiting for a vaccine injection. In our latest UK survey (Freeman et al., 2021), we therefore included assessment of blood-injection-injury fears in order to determine the degree to which they may be a factor in hesitancy about COVID-19 vaccination, which currently depends on injection.

There is a spectrum of severity of blood-injection-injury fears in the general population, with heritability estimated at approximately 40% (Van Houtem et al., 2013). Determined by diagnostic interview, lifetime prevalence of blood-injection-injury phobia in the adult population is approximately 3–4% (Stinson et al., 2007; Wardenaar et al., 2017). Rates are higher in women and in those of younger age. Self-identified fear of needles is much more common. One convenience sample study found that over 20% of parents and over 60% of children reported a fear of needles. Such fears were identified as the primary reason for immunisation non-compliance for 7% of parents and 8% of children (Taddio et al., 2012). Fear of injection has been cited by both the general public and health professionals as a reason for vaccination refusal, including for influenza, tetanus and pneumococcal infections (Johnson, Nichol, & Lipczynski, 2008; Yaqub, Castle-Clarke, Sevdalis, & Chataway, 2014). In a US survey conducted in June 2020, 11.8% of those who were hesitant about COVID-19 vaccination reported that a reason was a dislike of needles and injections (Ruiz & Bell, 2021). A higher proportion (43.8%) identified fear of dangerous side effects as the reason. In a US study of 9000 older adults conducted in November 2020, 1.7% were concerned about receiving a COVID-19 vaccine because of a fear of needles (Nikolovski et al., 2021). These studies suggest that, although fear of injection is not the dominant reason for vaccine hesitancy, it may be a contributory factor.

Our primary objective in this study was to determine the degree to which blood-injection-injury fears may explain COVID-19 vaccine hesitancy in the UK adult population. We also set out to estimate the prevalence of significant blood-injection-injury fears in the UK adult population. Furthermore, we wanted to test the extent to which injection fears may account for socio-demographic (age, gender, ethnicity and income) differences in COVID-19 vaccine hesitancy. Rather than merely asking a single question about fear of needles, we used established assessments. Our focus was on self-report, rather than diagnostic interview, since it is likely to be subjective thoughts and feelings across the spectrum of fear of injection that may have an impact regardless of whether they match diagnostic criteria. In a large survey of the adult general population, we included a self-report assessment of blood-injection-injury fears that can be used as a screening tool for the potential presence of a phobia. To validate results, we included an additional blood-injection fears questionnaire. We expected blood-injection-injury fears to explain a small amount of variance in vaccine hesitancy across the population. However, individuals with pronounced fears would be substantially more likely to be hesitant about taking a vaccine. We also hypothesised that injection fears may help explain (i.e. partially mediate) why vaccine hesitancy is somewhat higher in particular demographic groups (younger age, females, ethnic minority groups and lower income).

## Methods

### Participants

Oxford Coronavirus Explanations, Attitudes, and Narratives Survey (OCEANS) III (Freeman et al., 2021) is an online survey with a quota sampled UK participant group of 15 014 adults (18+ years old), conducted from 19 January 2021 to the 5 February 2021 via a market research company, Lucid. The quotas were based on UK Office for National Statistics population estimate data for gender, age, ethnicity, income and region. We did not wish to focus in this study only on those who had not been vaccinated by a particular point in time, since such results would be less generalisable, COVID-19 vaccination is highly unlikely to be a ‘one off’ event (there are two doses and it is likely to need renewal), and people who have been vaccinated still influence the rest of the population. Invited respondents did not know the topic of the survey before provisional agreement to complete it. They were simply told that there was a new survey and informed of the time period for it to be completed. Only after agreeing to participate did they see the online introduction. There was no mention of phobias in the rationale for the study provided, which was ‘There are now approved vaccines for COVID-19 that will be rolled out in the UK over the coming months. We want to learn about people's views about vaccination for COVID-19. In particular, we want to find out how many people would or would not wish to be vaccinated and the reasons behind their decision’. OCEANS-III was approved by the University of Oxford Central University Research Ethics Committee, and all participants provided informed consent online.

### Assessments

#### Initial vaccine hesitancy question

After agreeing to take part, participants completed a single question: ‘If the vaccine was available at my GP surgery I would: 1. Get it as soon as possible/2. Get it when I have time/3. Delay getting it/4. Avoid getting it for as long as possible/5. Never get it/6. Don't know’. This question was found in our OCEANS-II study to have the highest loading (0.95) on the COVID-19 vaccine hesitancy latent factor but for that precise reason was not included in the primary outcome measure (see Freeman et al., 2020 supplementary materials).

#### Oxford COVID-19 Vaccine Hesitancy Scale (Freeman et al., 2020)

Seven items [e.g. ‘Would you take a COVID-19 vaccine (approved for use in the UK) if offered?’] are rated on item specific response options (Saris, Krosnick, Revilla, & Shae, 2010), coded from 1 to 5. A ‘Don't know’ option is also provided, which is excluded from scoring. Higher scores indicate a higher level of vaccine hesitancy. In the current study the Cronbach's alpha of the scale was 0.97 (*N* = 15 014).

#### Specific Phobia Scale – blood-injection-injury (Ovanessian et al., 2019)

This is a self-report screening scale for different types of specific phobia based on the DSM-V criteria (APA, 2013). We used the 14 items assessing blood-injection-injury phobia. Each item (e.g. ‘Watching someone else get an injection’, ‘Getting minor surgery’, ‘Giving blood’ and ‘Receiving an injection’) is rated on a 5 point rating scale for the degree of fear triggered [0 (no fear) to 4 (extreme fear)]. Scores on the subscale can range from 0 to 56, with higher scores indicating a higher level of fear. A score of 20 or higher indicates the possible presence of a blood-injection-injury specific phobia (Ovanessian et al., 2019). In the current study, the Cronbach's alpha of the scale was 0.94 (*N* = 15 014).

#### Medical fear survey – short version – injections and blood (Olatunji et al., 2012)

Each of the four items [e.g. ‘Receiving a hypodermic (i.e. into the skin) injection in the arm’, ‘Having blood drawn from your arm’] is rated on a 4-point rating scale (0 = no fear to 3 = intense fear) for the degree of fear provoked. Scores can range from 0 to 12, with higher scores indicating greater fear. In the current study, the Cronbach's alpha of the scale was 0.90 (*N* = 15 014).

#### Injection fears

We also created from the two scales a factor score specifically for injection fears, using the four items about injection [‘Watching someone else get an injection’, ‘Receiving an injection’, ‘Receiving a hypodermic (i.e. into the skin) injection in the arm’, ‘Seeing someone receiving an injection in the arm’]. Higher scores indicate greater fear of injection.

### Statistical analysis plan

Statistical analyses were carried out in the R software environment for statistical computing and graphics, Version 4.0.3.

Structural equation modelling (SEM) was used to evaluate the factorial structure of the Oxford COVID-19 Vaccine Hesitancy Scale, Specific Phobia Scale, Medical Fear Survey and the combined four items measuring fear of injection, and to assess the potential mediation effect of fear of injection for associations between vaccine hesitancy and demographic factors. SEM is a collection of statistical techniques composed of two parts. The first part is the confirmatory measurement model, also known as the confirmatory factor analysis (CFA), which estimates the relations among latent constructs and their observed indicators. We assessed the measurement models of the existing and new injection fear scale based on factor loadings, inter-correlation between factors and several goodness-of-fit indices to ensure appropriate specification. The second part is the structural model, which estimates the relations among constructs (Kline, 2015). This combination allows the elimination of unreliability of measurement in the models and can be used to make assumptions about how latent constructs derived from observed variables are associated with each other. The mediation analysis was also conducted under the SEM framework and the advantages of such an approach are discussed in Gunzler, Chen, Wu, and Zhang (2013).

With non-normal distributions and missing values within the data, which in this case is considered either missing completely at random or missing at random due to test design (Little & Rubin, 2019; Rubin, 1976), we utilised the full information maximum likelihood estimation procedure with robust (Huber–White) standard errors and a scaled test statistic to estimate the SEMs, which has been described as a more efficient and less biased approach than other ad hoc missing data techniques (Enders, 2001; Enders & Bandalos, 2001). The SEM supports the data when the root mean square error of approximation (RMSEA) and standardised root mean square residual (SRMR) are <0.08 (Kline, 2005). Additional fit indices included the comparative fit index (CFI) and Tucker–Lewis Index (TLI), all of which should exceed 0.90 (Kline, 2015), with RMSEA < 0.06, and CFI and TLI > 0.95 indicating good model data fit (Hu & Bentler, 1999). The confidence intervals (CIs) of the indirect effects in the mediational model using SEM were tested using a Monte Carlo method with 20 000 replications as described by MacKinnon, Lockwood, and Williams (2004). The Holm method was used to adjust the *p* values in the mediational model using SEM to control the type I error rate due to multiple comparisons (Holm, 1979). The lavaan and the semTools R packages were used to conduct the SEM analyses (Jorgensen, Pornprasertmanit, Schoemann, & Rosseel, 2020; Rosseel, 2012).

We used the population attributable fraction (PAF) to evaluate vaccine hesitancy due to blood-injection-injury phobia. A historical review of the PAF is described by Poole (2015). The general idea of PAF is to understand the proportion of unfavourable outcomes that would have been prevented if the exposure of interest was eliminated from the population (Levin, 1953). Analysis of PAF was conducted using the AF R package (Dahlqwist, Zetterqvist, Pawitan, & Sjölander, 2016). We dichotomised (1, 2 = no hesitancy, 3, 4, 5 = hesitancy) the single vaccine hesitancy item that opened the survey (‘If the vaccine was available at my GP surgery I would…’). Participants who answered 6 (‘don't know’) were defined as missing and excluded from the analysis. The presence or absence of having a blood-injection-injury phobia was calculated based on the total score of the Specific Phobia Scale. Blood-injection-injury phobia was used as the exposure variable and was dichotomised according to the recommended cut-offs by Ovanessian et al. (2019). We used a logistic regression model to first estimate the log odds of vaccine hesitancy levels based on the presence of a phobia and included demographic associations (age, ethnicity and gender) as confounding controls. These demographic characteristics were known to be significant predictors as reported in OCEANS-II (Freeman et al., 2020). The beta coefficient estimates from the logistic regression model were then used to estimate the adjusted PAF with the formula described by Dahlqwist et al. (2016). The adjusted PAF was calculated as the proportion of vaccine hesitancy in the population that is attributable to blood-injection-injury phobia (i.e. the proportion of cases that might be prevented in the population if the exposure variable was eliminated), after accounting for demographic associations. The standard errors were estimated using the sandwich formula with the delta method (Greenland & Drescher, 1993; Sjölander & Vansteelandt, 2011).

## Results

A summary of the socio-demographic characteristics of the participants is provided in Table 1. For the initial vaccine hesitancy question – ‘If the vaccine was available at my GP surgery’ – 11 012 (73.3%) individuals reported that they would get it as soon as possible, 1451 (9.7%) reported that that they would get it when they have time, 672 (4.5%) reported that they would delay getting it, 771 (5.1%) reported that they would avoid getting it for as long as possible, and 632 (4.2%) said they would never get it. In total, 476 (3.2%) said that they did not know. Hence, 2075 (13.8%) participants were classed as hesitant (scoring 3, 4 or 5).

**Table 1.** Socio-demographic information (*N* = 15 014)

	Mean (s.d.)/*n* (%)
Age in years	47.2 (17.5)
Age ranges	
18–24	1770 (11.8%)
25–34	2579 (17.2%)
35–44	2473 (16.5%)
45–54	2634 (17.5%)
55–64	2233 (14.9%)
65–99	3325 (22.1%)
Gender: male; female; non-binary; prefer not say	7257 (48.3%); 7695 (51.3%); 41 (0.3%); 21 (0.1%)
Ethnicity	
White	
English/Welsh/Scottish/Northern Irish/British	11 988 (79.8%)
Irish	173 (1.2%)
Gypsy or Irish Traveller	44 (0.3%)
Any other White background	559 (3.7%)
Mixed/multiple ethnic groups	
White and Black Caribbean	136 (0.9%)
White and Black African	54 (0.4%)
White and Asian	128 (0.9%)
Any other mixed/multiple ethnic background	136 (0.9%)
Asian/Asian British	
Indian	364 (2.4%)
Pakistani	265 (1.8%)
Bangladeshi	148 (1.0%)
Chinese	130 (0.9%)
Any other Asian background	151 (1.0%)
Black/African/Caribbean/Black British	
African	321 (2.1%)
Caribbean	149 (1.0%)
Any other Black/African/Caribbean background	43 (0.3%)
Other ethnic group	
Arab	61 (0.4%)
Any other ethnic group	60 (0.4%)
Marital status	
Single	4626 (30.8%)
Married or civil partnership	7229 (48.1%)
Cohabiting	1818 (12.1%)
Separated	641 (4.3%)
Widowed	589 (3.9%)
Prefer not to say	111 (0.7%)
Highest level of education	
No qualifications	952 (6.4%)
GCSEs grades A*–C (or equivalent)	4148 (27.6%)
AS Levels (or equivalent)	621 (4.1%)
A Levels (or equivalent)	4068 (27.1%)
Certificate of higher education (e.g. BA, BSc, or equivalent)	3947 (26.3%)
Post graduate qualifications (e.g. MA, MSc, PhD, DPhil)	1276 (8.5%)
Total household income	
Less than £15 000	2348 (15.6%)
£15 000–19 999	1441 (9.6%)
£20 000–29 999	2768 (18.4%)
£30 000–39 999	2231 (14.9%)
£40 000–49 999	1584 (10.6%)
£50 000–59 999	1117 (7.4%)
£60 000–69 999	753 (5.0%)
£70 000–99 999	1049 (7.0%)
£100 000–149 999	539 (3.6%)
£150 000 and above	224 (1.5%)
Prefer not to say	960 (6.4%)
Housing situation	
Rented from council	2382 (15.9%)
Rented from private landlord	3039 (20.2%)
Homeowner	8738 (58.2%)
Other	855 (5.7%)
Region	
North East	636 (4.2%)
North West	1621 (10.8%)
Yorkshire and the Humber	1205 (8.0%)
East Midlands	1084 (7.2%)
West Midlands	1373 (9.1%)
East	1205 (8.0%)
London	1978 (13.2%)
South East	2177 (14.5%)
South West	1282 (8.5%)
Wales	750 (5.0%)
Scotland	1293 (8.6%)
Northern Island	410 (2.7%)
Pre-coronavirus employment status	
Unemployed	1168 (7.8%)
Employed full-time	6315 (42.1%)
Employed part-time	2061 (13.7%)
Self-employed	944 (6.3%)
Retired	3106 (20.7%)
Student	623 (4.1%)
Homemaker	797 (5.3%)
Had COVID-19	
Yes, a positive test	980 (6.5%)
No, a negative test	4519 (30.1%)
May have had it but not been tested	1620 (10.8%)
Not had it but not been tested	7695 (51.3%)
Other	200 (1.3%)
Risk for severe COVID-19 course	
Low risk	8608 (57.3%)
Moderate risk	4528 (30.2%)
Very high risk	1264 (8.4%)
Do not know	614 (4.1%)
Vaccinated for COVID-19	
No	13 139 (87.5%)
Yes, because in vulnerable group	806 (5.4%)
Yes, because in keyworker group	770 (5.1%)
Yes, for another reason	291 (1.5%)
Do not know	80 (0.5%)

### Prevalence of blood-injection-injury fears

There was a high correlation between fears as assessed with the Specific Phobia Scale and Medical Fears Survey, *r* = 0.81, *p* < 0.001, *n* = 15 014. The injection factor score correlated highly with the Specific Phobia Scale score, *r* = 0.89, *p* < 0.001, *n* = 15 014 and Medical Fears Survey score, *r* = 0.92, *p* < 0.001, *n* = 15 014. The mean score of the participant group on the specific phobia scale was 13.19, s.d. = 11.38, *n* = 15 014. In total, 3927 (26.2%) screened positive for a specific blood-injection-injury phobia and 11 097 (73.8%) screened negative.

### Creation of factor scores

Participants who had four or more missing items (i.e. answered don't know to over half the items) on the Oxford COVID-19 Vaccine Hesitancy Scale were removed from further analysis. This resulted in a sample size of 14 820 individuals, which was used for the CFA models. The CFA model for the Oxford COVID-19 Vaccine Hesitancy Scale indicated an excellent model fit (χ^2^ = 1112.84, df = 14, *p* < 0.001, CFI = 0.98, TLI = 0.97, RMSEA = 0.07, SRMR = 0.01). The initial CFA model indicated that the 14-item Specific Phobia Scale was unsatisfactory (χ^2^ = 11 328.93, df = 77, *p* < 0.001, RMSEA = 0.10, SRMR = 0.05, CFI = 0.86, TLI = 0.83). Therefore, in a post-hoc analysis, we evaluated the model adequacy based on the modification index (MI). MI is an estimate of the amount of chi-square which would be reduced if a parameter was added or the restriction of a specific parameter was removed in the CFA model (Sörbom, 1989). Specifically, we investigated MIs of correlated measurement errors between given pairs of items. Freely estimating covariances of correlated measurement errors with high MI results in a substantial decrease in the chi-square, thereby increasing model fit. However, the use of correlated measurement errors comes at an expense of theoretical accuracy since the covariance of the error term is from at least one unknown common source (Fornell, 1983; Gerbing & Anderson, 1984). It indicates that the specific pair of items has unexplained common sources of variance that are not captured by the latent variable. Given that the addition of the correlated measurement errors does not improve the interpretation of the model substantially, we instead used an alternative strategy, which was to remove one of the paired items that displayed high MI. We re-ran the analysis after the removal of each item to inspect the model's goodness-of-fit, which resulted in four items being removed (item 3, 8, 10, 14). The final model indicated acceptable goodness of fit results (χ^2^ = 2831.13, df = 35, *p* < 0.001, CFI = 0.93, TLI = 0.92, RMSEA = 0.07, SRMR = 0.04). The CFA model for the Medical Fear Survey indicated an excellent model fit (χ^2^ = 187.50, df = 2, *p* < 0.001, CFI = 0.99, TLI = 0.96, RMSEA = 0.08, SRMR = 0.02). The CFA model for the four-item injection fears subscale indicated an excellent model fit (χ^2^ = 174.98, df = 2, *p* < 0.001, CFI = 0.99, TLI = 0.96, RMSEA = 0.08, SRMR = 0.02).

### Associations of blood-injection-injury fears and vaccine hesitancy

The Oxford COVID-19 Vaccine Hesitancy Scale factor score correlated positively with the Specific Phobia Scale factor score, 0.22, *p* < 0.001, *n* = 14 820; the Medical Fear Survey factor score, *r* = 0.23, *p* < 0.001, *n* = 14 820; and the injection fears factor score, *r* = 0.25, *p* < 0.001, *n* = 14 820. (In a sensitivity analysis, the size of the associations was unchanged when controlling for condition in the OCEANS-III trial.) The individuals screening positive for a specific phobia were more likely to be hesitant (830/3754; 22.1%) on the survey opening hesitancy item (scoring 3, 4 or 5) than those screening negative for a specific phobia (1245/10 784; 11.5%), odds ratio = 2.18, 95% CI 1.97–2.40, *p* < 0.001.

### Explaining vaccine hesitancy and demographic associations

Mediation analysis using SEM was used to investigate the hypothesis that fear of injection partially mediates the effect of demographic associations on vaccine hesitancy. Hence, we tested a mediational model under the SEM framework consisting of age, gender, ethnicity and income. The sample size used in the analysis was *n* = 14 149. Participants who had missing data from the demographic variables and those who selected ‘other’ for gender (*n* = 32) were excluded. We also combined groups within ethnicity and income to increase the sample size at a group level. A breakdown of the demographic information is presented in Table 2.

**Table 2.** Socio-demographic information for mediation analysis (*N* = 14 149)

Demographic group	Group breakdown	Sample size (*n*)
Ethnicity	White	12 180
	Black	472
	White and other background	297
	Other mixed background	122
	Asian	969
	Other	109
Income	<20k	3445
	20k–40k	4799
	40k–60k	2607
	>60k	2490
	Prefer not to say	808
Gender	Male	6873
	Female	7276
Age		14 149

The mediation results are reported in Table 3. According to the mediation model, while holding demographic information constant, participants with a fear of injection had higher levels of vaccine hesitancy (*B* = 0.20, adjusted *p* < 0.01). The mediation model indicated that gender was not a significant predictor of fear of injection and vaccine hesitancy after controlling for age, ethnicity and income. Thus, further mediation analysis for gender was not conducted.

**Table 3.** Mediation results using SEM

	Effect	s.e.	Lower CI	Upper CI	*β*	Adjusted *p* value
(Relative) effect of IV on mediator (fear of injection)						
Age	−0.015	0.000	−0.016	−0.015	−0.344	0.003
Black	0.208	0.040	0.129	0.287	0.048	0.003
White Other	0.140	0.055	0.032	0.248	0.026	0.132
Other mixed background	−0.005	0.077	−0.156	0.146	−0.001	1.000
Asian	0.168	0.029	0.110	0.225	0.054	0.003
Other background	0.160	0.080	0.003	0.317	0.018	0.450
20k to 40k	−0.033	0.017	−0.067	0.001	−0.020	0.450
41k to 60k	−0.059	0.020	−0.099	−0.019	−0.029	0.060
>60k	−0.096	0.021	−0.137	−0.055	−0.047	0.003
Prefer not to say income	0.003	0.032	−0.060	0.065	0.001	1.000
Gender	0.038	0.013	0.013	0.064	0.024	0.060
Unique effect of mediator on outcome (hesitancy score)						
Fear of injection	0.203	0.013	0.177	0.229	0.166	0.003
(Relative) indirect effect						
Age	−0.003	0.000	−0.004^a^	−0.003^a^	−0.057	0.003
Black and fear of injection	0.042	0.009	0.026^a^	0.059^a^	0.008	0.003
White other and fear of injection	0.028	0.011	0.006^a^	0.051^a^	0.004	0.132
Other mixed background and fear of injection	−0.001	0.016	−0.031^a^	0.030^a^	0.000	1.000
Asian and fear of injection	0.034	0.006	0.022^a^	0.047^a^	0.009	0.003
Other background and fear of injection	0.032	0.016	0.001^a^	0.065^a^	0.003	0.450
Income between 20k to 40k and fear of injection	−0.007	0.004	−0.014^a^	0.000^a^	−0.003	0.450
Income between 41k to 60k and fear of injection	−0.012	0.004	−0.020^a^	−0.004^a^	−0.005	0.060
Income >60k and fear of injection	−0.020	0.004	−0.029^a^	−0.011^a^	−0.008	0.003
(Relative) direct effect of IV on outcome (hesitancy score)						
Age	−0.013	0.001	−0.014	−0.012	−0.241	0.003
Black	0.700	0.053	0.595	0.805	0.131	0.003
White other	0.221	0.063	0.097	0.345	0.033	0.003
Other mixed background	0.357	0.104	0.153	0.560	0.034	0.016
Asian	0.140	0.032	0.076	0.204	0.037	0.003
Other background	0.246	0.096	0.059	0.433	0.022	0.003
20k to 40k	−0.112	0.021	−0.154	−0.070	−0.055	0.003
41k to 60k	−0.249	0.024	−0.295	−0.203	−0.100	0.003
>60k	−0.301	0.025	−0.350	−0.252	−0.119	0.003
Prefer not to say income	−0.072	0.036	−0.141	−0.002	−0.017	0.450
Gender	−0.002	0.015	−0.032	0.028	−0.001	1.000
(Relative) total effect						
Indirect effects and direct effects	1.011	0.194	0.630	1.392	−0.325	0.003

CI, confidence intervals; s.e., standard error.

a Monte Carlo CI. Reference group for ethnicity: White. Reference group for income: less than 20k. Adjusted *p* values using the Holm method.

Age was a significant predictor of fear of injection (*B* = −0.015, adjusted *p* < 0.01) and the indirect effect of age and vaccine hesitancy via fear of injection was also found to be significant (*B* = −0.003, adjusted *p* < 0.01). Age remained a significant predictor of vaccine hesitancy after controlling for fear of injection (mediator), indicating a partial mediation (*B* = −0.013, adjusted *p* < 0.01).

The mediation for ethnicity indicated that relative to White ethnicity, only Black and Asian ethnicities were significant predictors of fear of injection (*B* = 0.208, adjusted *p* < 0.01; *B* = 0.168, adjusted *p* < 0.01). The relative indirect effects between ethnicity and vaccine hesitancy via fear of injection were also found to be significant only for Black and Asian ethnicities (*B* = 0.042, adjusted *p* < 0.01; *B* = 0.034, adjusted *p* < 0.01). The relative direct effects between ethnicity and vaccine hesitancy remained significant for Black and Asian ethnicities (*B* = 0.70, adjusted *p* < 0.01; *B* = 0.14, adjusted *p* < 0.01) after controlling for the fear of injection, indicating a partial mediation for these ethnic groups. Relative to White ethnicity, White other, Other mixed background and Other ethnic groups were also associated with higher levels of vaccine hesitancy (*B* = 0.22, adjusted *p* < 0.01; *B* = 0.36, adjusted *p* < 0.05; *B* = 0.25, adjusted *p* < 0.01), but the relationships between these ethnic groups and vaccine hesitancy were not (partially) mediated by fear of injection.

**Fig. 1.**
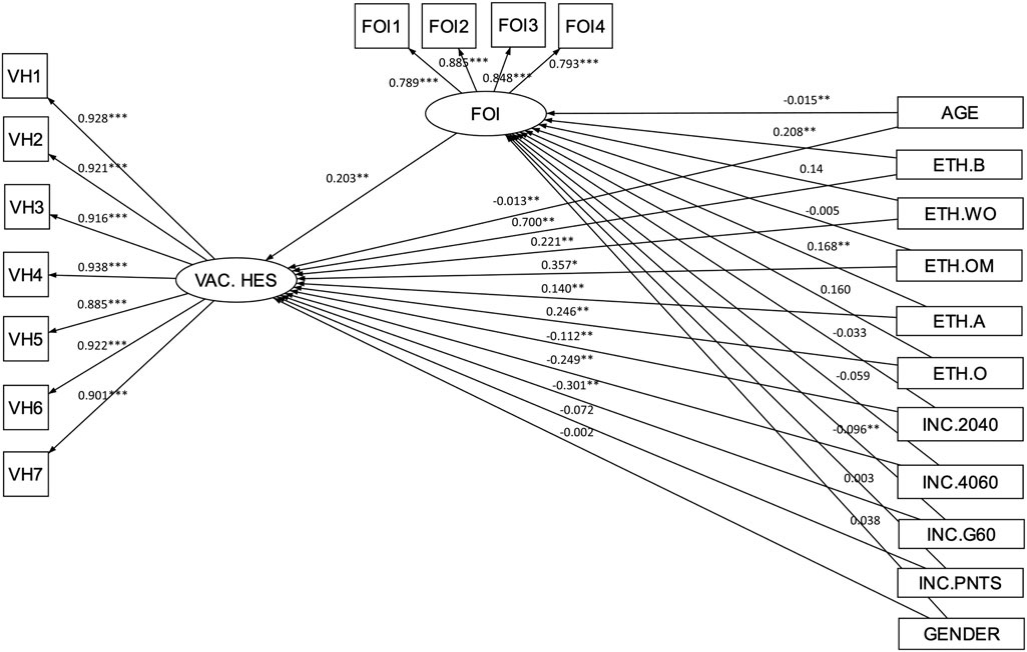
Mediational model using SEM for demographic associations with vaccine hesitancy. FOI, fear of injection; VAC.HES, vaccine hesitancy; ETH.B, Black ethnicity; ETH.WO, White other ethnicity; ETH.OM, other mixed background ethnicity; ETH.A, Asian ethnicity; ETH.O, other ethnicity; INC.2040, income group between £20k and £40k; INC.4060, income group between £40k and £60k; INC.G60, income group greater than £60k; INC.PNTS, prefer not to say income group. Reference group for ethnicity is White. Reference group for gender is male.

Relative to the income group with less than £20k, only those in the greater than £60k income group was a significant predictor of fear of injection (*B* = −0.096, adjusted *p* < 0.01). The relative indirect effect between those in the greater than £60k income group and vaccine hesitancy via fear of injection was also significant (*B* = −0.02, adjusted *p* < 0.01). The relative direct effect for the greater than £60k income group remained significant (*B* = −0.30, adjusted *p* < 0.01) after controlling for the fear of injection, indicating a partial mediation. Relative to the less than £20k income group, those in the £20k to £40k and £41k to £60k income groups were associated with lower levels of vaccine hesitancy (*B* = −0.11, adjusted *p* < 0.01; *B* = −0.25, adjusted *p* < 0.01), but the relationships were not (partially) mediated by fear of injection. The relative direct effects for those in the Prefer not to say income group were not significant for both vaccine hesitancy and fear of injection. Thus, further mediation analysis for the Prefer not to say income group was not necessary. The total effect combining all (relative) direct and indirect effects was significant (*B* = 1.011, adjusted *p* < 0.01). Approximately 16.1% (*R*^2^ = 0.161) of the variance in vaccine hesitancy was accounted for by the demographic variables in the mediation model. The model showed an excellent fit (χ^2^ = 2701.92, df = 142, *p* < 0.001, RMSEA = 0.04, SRMR = 0.01, CFI = 0.98, TLI = 0.97). Figure 1 presents the mediational model using SEM.

### Population attributable fraction

**Fig. 2.**
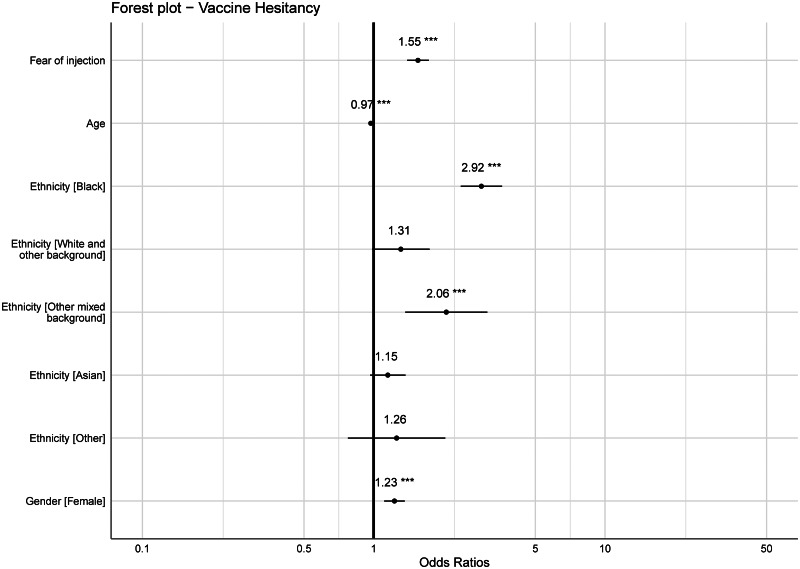
Forest plot of associations with vaccine hesitancy (all factors included in the same model). Reference group for gender is male and for ethnicity is White.

The PAF analysis was conducted based on a sample size of *n* = 13 875 after removing participants who answered ‘don't know’ to the initial vaccine hesitancy item and those with missing responses to the demographics information, including those who answered ‘other’ to gender. According to the logistic model, the log of the odds of people being vaccine hesitant was positively related to the presence of blood-injury-injection phobia (*B* = 0.44, s.e. = 0.06, 95% CI 0.33–0.55, *p* < 0.001). The odds for participants with a blood-injection-injury phobia being vaccine hesitant were 1.55 ( = *e*^0.4409^) times greater than the odds for participants with no blood-injection-injury phobia. The estimated PAF indicated that approximately 11.5% of all participants who are vaccine hesitant might have been prevented if fear of injection was absent (AF = 0.1149; s.e. = 0.02, 95% CI 0.09–0.14, *p* < 0.001). The odds ratios for different factors associated with vaccine hesitancy are summarised in a forest plot generated from a single model that contained all factors (see Fig. 2).

## Discussion

Blood-injection-injury fears are remarkably common in the UK adult general population. Our findings clearly show that these fears have a small to medium association with COVID-19 vaccine hesitancy. Approximately 5% of variance in COVID-19 hesitancy scores across the adult population may be accounted for by injection fears. At an individual level, screening positive for a blood-injection-injury phobia doubles the odds of also reporting COVID-19 vaccine hesitancy. Around a quarter of the population screen positive for potentially having such a phobia. A PAF calculation indicated that if blood-injection-injury phobia were eliminated, then just over 10% of instances of people who are COVID-19 vaccine hesitant might also be removed. It was also found that two demographic factors repeatedly found to be associated with vaccine hesitancy (age and ethnicity) were explained to a small degree by higher levels of injection fears in these groups. Overall, the results indicate that fear of injection may have a small role to play in the occurrence of vaccine hesitancy in adults. It is a highly plausible causal connection, and one which we find is voiced by individuals who are vaccine hesitant. However, the key caveat of this study is that all the analyses were correlational, and causation cannot be determined. Only future studies that lessen fears of injection and test effects on vaccine hesitancy can determine causation with certainty.

Our survey used a non-probability online quota sampling method, which will have introduced bias into who was approached to take part, and hence caution is required particularly for prevalence estimates. Injection fears will also be higher in children. Given the plausible causal connection, however, in our view the study findings indicate that injection fears should be addressed in vaccination programmes. The fewer the barriers to vaccine acceptance, the more successful the programme. Fear of injection is likely to be a barrier for some individuals but it can be addressed in a number of ways. First, alternative needle-free delivery routes of COVID-19 vaccination could be made available. For example, there has been a nasal spray developed for childhood flu and oral vaccines developed for rotavirus and for polio. Second, administration of the vaccine by injection could be made more tolerable, producing positive narratives in the general public of the process. For example, looking away from the injection may reduce fear (Mithal et al., 2018). Pain mitigation strategies (e.g. acute exercise) may also be helpful (Edwards & Booy, 2019). Third, there could be wider provision of the brief psychological treatments (exposure therapy and applied tension) for blood-injection-injury fears that show large treatment effects (Ayala, Meuret, & Ritz, 2009; Hiermeier & Mofrad, 2020; McMurtry et al., 2016). The success of vital COVID-19 vaccination programmes is dependent on uptake. As such, we should not underestimate the importance of making the thought of the jab less anxiety-provoking for the millions of people who are fearful of injections.

## Supplementary material

For supplementary material accompanying this paper visit http://dx.doi.org/10.1017/S0033291721002609. click here to view supplementary material
