# Supplementary material for: Injection fears and COVID-19 vaccine hesitancy
Source: Psychol Med. 2021 Jun 11:1–11. doi: 10.1017/S0033291721002609 (PMC8220023; doi:10.1017/S0033291721002609)
Supplement: Supplementary file 1 [file S0033291721002609sup001.docx]

**Supplementary materials**

The software and method used to conduct confirmatory factor analysis are described in the Statistical Analysis Plan section of the main article. The tables below present the factor loadings of the individual scales. The goodness-of-fit indices are reported in the main article.

Table S1. Standardised loadings based on confirmatory factor loadings (Vaccine Hesitancy Scale)

| Item | Factor loading |
| --- | --- |
| Item 1 (C1) | 0.93 |
| Item 2 (C2) | 0.92 |
| Item 3 (C3) | 0.92 |
| Item 4 (C4) | 0.94 |
| Item 5 (C5) | 0.89 |
| Item 6 (C6) | 0.92 |
| Item 7 (C7) | 0.90 |
| *Note*. Standardised item loadings for a single factor. | |

Table S2. Standardised loadings based on confirmatory factor loadings (Specific Phobia Scale)

| Item | Factor loading |
| --- | --- |
| Item 1 (F1r1) | 0.75 |
| Item 2 (F1r2) | 0.74 |
| Item 4 (F1r4) | 0.67 |
| Item 5 (F1r5) | 0.79 |
| Item 6 (F1r6) | 0.66 |
| Item 7 (F1r7) | 0.49 |
| Item 9 (F1r9) | 0.64 |
| Item 11 (F1r11) | 0.72 |
| item 12 (F1r12) | 0.83 |
| Item 13 (F1r13) | 0.55 |
| *Note*. Standardised item loadings for a single factor. | |

Table S3. Standardised loadings based on confirmatory factor loadings (Medical Fear Survey)

| Item | Factor loading |
| --- | --- |
| Item 1 (G1r1) | 0.87 |
| Item 2 (G1r2) | 0.89 |
| Item 3 (G1r3) | 0.80 |
| Item 4 (G1r4) | 0.78 |
| *Note*. Standardized item loadings for a single factor. | |

Table S4. Standardised loadings based on confirmatory factor loadings (Injection Fears Survey)

| Item | Factor loading |
| --- | --- |
| Item 1 (F1r1) | 0.79 |
| Item 2 (F1r12) | 0.88 |
| Item 3 (G1r1) | 0.85 |
| Item 4 (G1r4) | 0.79 |
| *Note*. Standardised item loadings for a single factor. | |
